# P-517. Symptom Severity and Functional Impact of Medically-Attended Influenza, SARS-CoV-2 and RSV Illnesses among Children

**DOI:** 10.1093/ofid/ofaf695.732

**Published:** 2026-01-11

**Authors:** Oluwakemi Alonge, Huong Q Nguyen, Gigi Zheng, Jennifer P King, Wen-Hsing Wu, Meng Wang, Emma Viscidi, Catherine A Panozzo, Chelsea Canan, Shivani Nagapurkar, Jennifer K Meece, Evan J Anderson, Joshua Petrie

**Affiliations:** Marshfield Clinic Research Institute, Marshfield, WI; Marshfield Clinic Research Institute, Marshfield, WI; Moderna, Inc., Cambridge, Massachusetts; Marshfield Clinic Research Institute, Marshfield, WI; Moderna, Inc., Cambridge, Massachusetts; Moderna, Inc., Cambridge, Massachusetts; Moderna Therapeutics, Cambridge, Massachusetts; Moderna, Inc., Cambridge, Massachusetts; Moderna, Inc., Cambridge, Massachusetts; Marshfield Clinic Research Institute, Marshfield, WI; Marshfield Clinic Research Institute, Marshfield, WI; Moderna, Inc., Cambridge, Massachusetts; Marshfield Clinic Research Institute, Marshfield, WI

## Abstract

**Background:**

Respiratory illnesses resulting from influenza, respiratory syncytial virus (RSV), and SARS-CoV-2 share overlapping symptoms and pose a significant public health burden, particularly in young children. We assessed symptom severity and functional impact of medically attended influenza, RSV and SARS-CoV-2 acute respiratory illness (ARI) among children.

**Table.**
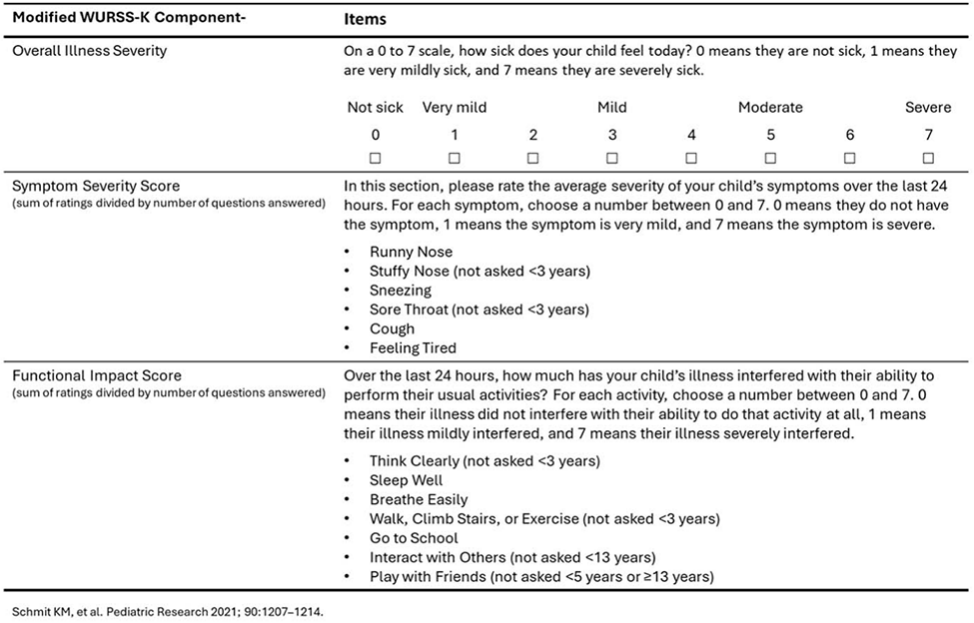
Overview of Modified Wisconsin Upper Respiratory Symptom Survey – Kids (WURRS-K).

**Methods:**

Patients aged < 18 years with ARI were enrolled from inpatient and outpatient settings and tested for influenza, RSV, and SARS-CoV-2 between September 2024 and March 2025. We used a modified Wisconsin Upper Respiratory Symptom Survey for kids (WURSS-K) instrument to assess symptom severity, functional impact, and overall illness severity (see table for details on WURSS-K score calculation). WURSS-K scores were compared across each virus in multiple linear regression models adjusted for age group, presence of ≥1 chronic condition, and days from illness onset to enrollment.

**Results:**

Among 1,055 children enrolled, 123 (12%), 102 (9.6%) and 40 (3.7%) tested positive for influenza, RSV and SARS-CoV-2, respectively; 11 participants with co-detection of ≥1 of these 3 viruses were excluded from further analysis. Among 977 enrolled in the outpatient setting, 117 (12%) were positive for influenza, 75 (8%) for RSV and 38 (4%) for SARS-CoV-2. Among 67 inpatients, 6 (9%) were positive for influenza, 27 (40%) for RSV and 2 (3%) for SARS-CoV-2. The median (interquartile range) age was 8 (4, 13) years for influenza, 2 (1, 6) years for RSV, and 4 (0.5, 12) years for SARS-CoV-2 cases. The adjusted mean (95% CI) symptom severity score for influenza (4.2 [3.9, 4.5]) was higher than SARS-CoV-2 (3.4 [2.9, 3.8]; p=0.009), but similar to RSV (3.9 [3.6, 4.3]; p=0.14). The adjusted mean (95% CI) functional impact score for influenza (4.2 [3.9, 4.5]) was higher than RSV (3.8 [3.4, 4.1]; p=0.04), but similar to SARS-CoV-2 (3.9 [3.5, 4.5]; p=0.4). Adjusted overall illness severity score was generally mild to moderate, but influenza (4.7 [4.5, 4.9]) was higher than RSV (4.2 [4.0, 4.6]; p=0.03) and SARS-CoV-2 (4.1 [3.6, 4.5]; p=0.01) among children of all ages.

**Conclusion:**

In 2024-2025, among mostly outpatient children, the highest WURSS-K score was noted for influenza; however, absolute differences between influenza, RSV and SARS-CoV-2 were relatively small.

**Disclosures:**

Oluwakemi Alonge, MPH, CPH, CSL Seqirus: Grant/Research Support|GSK: Grant/Research Support|ModernaTX: Grant/Research Support Huong Q. Nguyen, PhD, MPH, CSL Seqirus: Advisor/Consultant|CSL Seqirus: Grant/Research Support|GSK: Grant/Research Support|ModernaTX: Advisor/Consultant|ModernaTX: Grant/Research Support Gigi Zheng, MD, PhD, ModernaTX: Employee|ModernaTX: Stocks/Bonds (Public Company) Jennifer P. King, MPH, GSK: Grant/Research Support|ModernaTX, Inc.: Grant/Research Support Wen-Hsing Wu, MS, Moderna: Stocks/Bonds (Public Company) Meng Wang, PhD, ModernaTX: Employee|ModernaTX: Stocks/Bonds (Public Company) Emma Viscidi, PhD, MHS, ModernaTX: Employee|ModernaTX: Stocks/Bonds (Public Company) Catherine A. Panozzo, PhD, ModernaTX: Employee|ModernaTX: Stocks/Bonds (Public Company) Chelsea Canan, PhD, ModernaTX: Employee|ModernaTX: Stocks/Bonds (Public Company) Shivani Nagapurkar, MPH, ModernaTX: Grant/Research Support Jennifer K. Meece, PhD, CSL Seqirus: Grant/Research Support|GSK: Grant/Research Support|ModernaTX: Grant/Research Support Evan J. Anderson, MD, Moderna: Stocks/Bonds (Public Company) Joshua Petrie, PhD, CSL Seqirus: Advisor/Consultant|CSL Seqirus: Grant/Research Support|ModernaTX: Grant/Research Support

